# On the Waves of the COVID-19 Pandemic

**DOI:** 10.3390/jcm12041338

**Published:** 2023-02-08

**Authors:** Michele Roccella, Antonio Fallea, Luigi Vetri

**Affiliations:** 1 Department of Psychology, Educational Science and Human Movement, University of Palermo, 90128 Palermo, Italy; 2 Oasi Research Institute-IRCCS, Via Conte Ruggero 73, 94018 Troina, Italy

The COVID-19 pandemic has been a tsunami that has deeply changed the lives of the people all over the planet. Today, the pandemic is at a stage where we have gained enough experience to be able to begin to look beyond its immediate effects. In fact, although the spread of new COVID-19 variants is proceeding at a unprecendented pace, we have entered a new phase of the epidemic cycle where, once the isolation and “escape” phase from the virus has ended, we can focus on coexistence with the new variants. This is also thanks to public health measures, especially the introduction of vaccines that have drastically reduced the rates of hospitalization and mortality [1,2].

At a time when governments of various states around the world are establishing new frameworks toward a new normal, it is important that scientific research focuses on new and more effective measures to fight the pandemic and, at the same time, proceed to analyze the impact that the pandemic has had on people’s quality of life and psychophysical well-being [3,4]. 

A total of six studies, five research articles and one systematic review are included in the Special Issue “The Impact of the COVID-19 Emergency on the Quality of Life of the General Population, Part II”. These papers analyze the secondary neuropsychological effects of the COVID-19 pandemic. 

In their study, Rogowska et al. analyzed the physical and mental well-being of Polish and Ukrainian university students during the second wave of the COVID-19 pandemic through standardized questionnaires on life satisfaction, anxiety, depression, physical health, perceived stress and coronavirus-related PTSD. The authors found increased levels of stress, anxiety and depression anddecreased life satisfaction and physical health. The authors highlighted that low perceived COVID-19-related stress directly influenced life satisfaction and anxiety level [5]. 

Similarly, García-Garro et al., in their longitudinal study, investigated changas in depression levels, quality of life, quality of sleep, and physical activity in Colombian university students during the COVID-19 pandemic. The authors administered the Zung Self-Rating Depression Scale, the International Physical Activity Questionnaire, the SF-12v2 and the Pittsburgh Sleep Quality Index through an online survey and showed low quality of life, decreased quality of sleep and increased depression symptoms, especially during mandatory confinement periods [6].

In addition to this evidence, Kupcewicz et al. underlined that a positive orientation, intended as a combination of self-esteem, optimism and life satisfaction, influenced the tiredness/fatigue experienced by a sample of nursing students. Students with major positive orientation manifested lower levels of tiredness/fatigue. Therefore, the authors suggested the implementation of preventive measures to intensify positive orientation and therefore minimize the negative effects of the COVID-19 pandemic [7].

Furthermore, Gago-Valiente et al. evaluated the mental health of healthcare professionals using internationally validated instruments and assessed that nursing assistants having high contact with COVID-19-positive patients had poor mental health indicators in terms of emotional exhaustion, depersonalization and probable non-psychotic psychiatric pathologies. Interestingly, the authors found significative gender differences related to emotional exhaustion and depersonalization that were more prevalent in men than in women. These data underline that gender differences must be taken into account when implementing public health measures to defend the neuropsychological well-being of workers in high-risk categories [8]. 

Originally, Rupp et al. tried to evaluate the influence of genetic components on pandemic-associated fatigue. They administered the Multidimensional Fatigue Inventory to 55 monozygotic and 45 dizygotic twin pairs highlighting that fatigue symptoms were significantly similar within twin pairs; on the contrary, the intensity and the severity of pandemic-associated fatigue dimensions were found to be mainly influenced by individual environmental factors, such as the number of comorbidities one has [9]. 

Several studies confirm beyond any doubt that the COVID-19 pandemic has had a negative impact on people’s psychophysical well-being, but an open question is which measures are more appropriate to put in place to contrast these negative effects. D’Onofrio et al., in their systematic review and meta-analysis, evaluated the effectiveness of psychological interventions used during the SARS-COV-2 pandemic, analyzing psychological improvements in terms of the reduction in depression, anxiety and post-traumatic stress symptoms. Their analysis demonstrated that, globally, mental health scores were significantly better compared to the pre-intervention scores, and in particular, cognitive behavioral therapy had the greatest effect size on the mitigation of psychological impairment and on increasing resilience [10]. 

The world has faced several epidemic waves since the beginning of the COVID-19 pandemic. In order to for a wave of any type to occur, two elements must be present: a factor that increases the epidemic and something that decreases it. Infectious studies underline that the main element determining the increase in epidemic waves is the contagiousness of the infectious agent together with the vulnerability of those exposed to the contagion. On the contrary, the elements contrasting the epidemic wave are decreased contagiousness, lower susceptibility and containment measures put in place [11,12]. What infectious studies sometimes overlook is that between one wave and another and after that last wave passes, what will remain will be a series of pandemic-related issues, even if not directly caused by the virus. Among them, as the studies collected in this Special Issue confirm, neuropsychological sequelae are among the most fearsome and pressing and require urgent measures of public health to prevent their onset and mitigate their effects.
